# Being a scientist

**DOI:** 10.1038/s41430-022-01079-5

**Published:** 2022-02-01

**Authors:** Manfred J. Müller

**Affiliations:** Christian-Albrechts-Universität zu Kiel, Düsternbrooker Weg 17-19, D-24105 Kiel, Germany

**Keywords:** Education, Nutrition


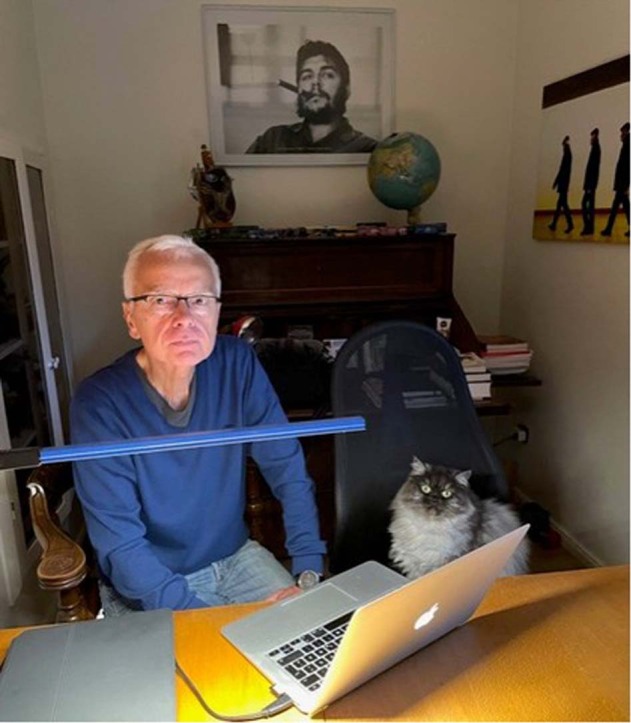

*Can one tell the time, this itself as such, in and of itself? Really, no, that would be a foolish endeavor. (from Thomas Mann, The Magic Mountain)*.

The begin of my career as a scientist had been in the first part of the 70s of the last century. Thus, I now have nearly 50 years of experience in biomedical research. I had started in a department of biochemistry in Hamburg, went on into various areas of clinical work (i.e., endocrinology, intensive care medicine, gastroenterology, and clinical nutrition) in Hannover and Geneva, took an intermediate step working in a Federal Health Office in Berlin and finally ended up as a full professor of Human Nutrition in Kiel, where I had worked for the last 23 years. As for many of my colleagues, becoming an expert in clinical nutrition was more or less coincidental for me. My major research focus was and still is on energy metabolism while during the last decades I also became interested in certain aspects of public health nutrition (e.g., prevention of childhood obesity and planetary health).

Today, the appreciation of a scientist mostly depends on (i) the number of his or her papers published in journals characterized by high impact factors, (ii) the successful acquisition of research money from grants and funding agencies, (iii) the number of given awards, (iV) being part of a network of reputed scientists as well as (V) the position reached at a (more or less) outstanding institution. Although I can refer to more than 650 publications, an appropriate funding, a respectable network, and a good academic position, I do not consider these criteria as essential. Alternatively, being a scientist is about a life-long step-by-step approach ending up with a slightly improved understanding of his or her science.

Understanding is about a general idea rather than about details (which again may have more or less short half-lives).

## Looking back: some insights from my research activities

Some insights from my research work are described below, they may provide food for thought and thus may add to the discussion. Most of my own research was about fuel metabolism during catabolism at the whole body and the organ/tissue level. To name a few authors my ideas and research questions mainly followed some seminal contributions published by Keys [1], Krebs and Veech [2], Cahill [3], Felig et al. [4].

- Within the first hours after a meal, changes in glucose, lipid and energy metabolism (i.e., the metabolic fluxes) do not coincide with changes in the amounts of key enzyme (e.g., hepatic phosphofructokinase and pyruvate kinase activities; [5, 6]). In addition, measuring serum and tissue concentrations of intermediates of a metabolic pathway without a concomitant assessment of the specific metabolic fluxes (or at least the net balances across organs, tissues, body regions and/or the whole body) does not allow any deeper insights into metabolic control [7, 8].
- Thyroid hormones have a pivotal role regulating intermediary metabolism, i.e., they may have an anabolic or a catabolic effect depending on energy balance. For example, with overfeeding an increase in T3 increases hepatic lipogenesis whereas the same level of T3 increases ketogenesis during fasting [9–11].
- From a metabolic point of view, insulin resistance is not a unique phenotype. For example, patients with hyperthyroidism, liver cirrhosis, and diabetes differ from each other with respect to the specific disturbances in oxidative and non-oxidative glucose metabolism [12, 13]. Concomitantly, all these diseases show an exaggerated response to stress (e.g., exaggerated responses to increased SNS activity and elevated glucagon levels) favoring tissue catabolism [14–17]. A low insulin level and/or insulin resistance add to explain this phenomenon [18].
- Increased energy expenditure and metabolic disturbances seen in patients with liver cirrhosis reflect an extrahepatic manifestation of liver disease (i.e., after manifestation they are in part independent of the clinical and biochemical characteristics of liver disease) which persists for more than one year after liver transplantation [19–21].
- Individual organ and tissue metabolism differ from whole-body metabolism, e.g., during starvation liver catabolism exceeds whole body catabolism while the hepatic respiratory exchange ratio (RQ) exceeded whole body RQ with overfeeding [22, 23].
- Characterizing individual body components rather than body mass itself provides an alternative basis for metabolic phenotyping. For example, detailed body composition at the anatomical and/or molecular level can explain up to 80% of the inter-individual variance in resting energy expenditure (REE) as well as the non-zero intercept of the REE on fat free mass-regression line [24, 25]. By contrast, individual body components have a limited precision to predict cardio-metabolic risks [26, 27].
- The concept of ‘functional body composition’ (FBC) provides an interpretation of body functions and their disturbances [28–30]. Vice versa, FBC contributes to explaining the meaning of individual body components. FBC is a ‘multilevel–multi-systemic approach’ to address the associations (i) between masses of organs and tissues, (ii) between these masses and their respective metabolic functions, (iii) within the contexts of neurohumoral control and finally (iv) in relation to ‘systemic’ outcomes, e.g., on body temperature, heart rate, glomerular filtration rate, and respiration.
- Metabolic adaptation to starvation is a dynamic process including early (within hours and the first days), intermediate (up to weeks) and late responses (during chronic caloric restriction) which differ with respect to changes in body composition and in the endocrine determinants of metabolism [31–34]. Adjusting the decrease in REE with underfeeding for the corresponding changes in FFM as well as it’s anatomical and molecular composition metabolic adaptation was likely not large enough in magnitude to be able to prevent weight loss questioning the narrative of a thrifty and weight loss-resistant phenotype [35].
- Differences in energy turnover (e.g., due to different intensities of physical activity or a different contribution of organ and tissue masses during cold-exposure, growth, pregnancy, lactation or high protein intake) exert discrepant metabolic effects that impact energy intake and energy balance [36–38].
- In industrialized countries, prevention of obesity has limited success only [39–41]. This is because we widely ignore that the issue has to be seen within broader and international contexts, i.e., to find a solution of the obesity issue societal, economical as well as ecological aspects have to be taken into account [42]. As has been proposed by the recent report of the Lancet Obesity Commission, obesity is the result of “many manmade systems” (including the food system) which have to be “re-oriented” for better population health [43].

## Looking forward: a critical view at today’s research activities

While remembering the past, looking into the crystal ball also gives rise to consider some recent and future developments in biomedical research. When compared to today’s life of a scientist my first mentor had made different experiences, i.e., he had one grant per time and published two papers per year ‘only’. By contrast, today’s research has become a flourishing business with guaranteed profits in terms of more and more papers (e.g., our top researchers or so-called ‘academic heroes’ usually publish more than 50 papers per year) which again serve as a suitable basis of raising further money for the benefits of the scientists themselves, their institutions as well as the publishers of scientific journals. Presently, it may look like that some top scientists have become steadfast, insatiable, and more and more successful. Thus, science may have become a never-ending ‘success story’.

As in every successful business model, we have to be aware of certain systemic side effects of too much economic growth and success which may arouse further desires. The risks of modern biomedical research (which have been already addressed in more detail before, see ref. [44, 45]) include

- rushing the time of research driven by the idea that all the big biomedical issues have to be solved within short-term,
- trusting too much on certain narratives (e.g., on the mismatch between our genetic predisposition and modern lifestyles as cause of non-communicable diseases; NCD) which have never been proven in detail,
- ignorance of a public health point of view (e.g., proposing that individualized strategies like precision nutrition are a solution for population-wide diseases like obesity and NCD),
- lack of defining appropriate phenotypes worthwhile to address by the use of modern biomedical methods and techniques,
- careless applications of modern technologies and methods (e.g., using metabolomics in serum and urine samples without taking into account methodological issues of probe sampling and the stability and half-lives of individual metabolites),
- following merely the reductionistic approaches of molecular and cellular biology losing sight of clinical and solution-oriented research,
- data driven research rather than sound and hypotheses-based strategies,
- data analyses depending on artificial intelligence rather than on basic knowledge and plausibility,
- uncritical use of statistics (e.g., a statistical significance is frequently used as an equivalent of scientific inference) [46, 47],
- the occurrence of avoidable mistakes up to fraud in science [48–50] and
- the establishment of new hierarchical structures in our communities and institutions where so-called ‘successful’ scientists take over the power (i.e., they also decide about the future of science and the distribution of research money).

Throwing a critical (and may be a too critical) eye on today’s high productivity in research (and thus the incredibly high rate of publishing) it may look like that the system has become more or less out of control. For example, faced with the presently high number of nutrition journals and, thus, a long list of articles published each month I would assume that today no colleague has time for such endless reading anymore. This scenario would suggest a certain loss of knowledge and the end of a broad discussion between scientists.

However, control has to be regained within our scientific communities, i.e., by the scientists themselves. It is obvious, that there is an urgent need of self-critical discussions about what we are presently doing within and between the communities of scientists. To start with, it would be helpful for all of us to accept that science is not special, it is simply about the “not knowing” (which is challenging to address, no question about that). This in mind we may come down a little bit on the carpet again.

In addition, it is worthwhile to accept the finiteness of research. As the Oxford philosopher Mary Midgley has already said, ‘Science is a process to obtain as true or approximate true understanding of the real world’ but it is deceptive optimism to assume that ‘Science and technology will answer all our questions and solve all our problems’ [51]. Thus, without taking an “anti-science” point of view, too much appreciation of science may be seen as a dogma with a repressive effect [52]. To stimulate a forthcoming discussion about values of modern biomedical research I would like to refer to Hannah Arendt’s critical view on truth in modern sciences: ‘The trouble concerns the fact that the truths of the modern scientific world view, though they can be demonstrated in mathematical formulas and proved technologically, will no longer lend themselves to normal expression in speech and thought…In this case, it would be as though our brain, which constitutes the physical, material condition of thoughts, were unable to follow what we do, so that from now on we would indeed need artificial machines to do our thinking and speaking. If it should turn out to be true that knowledge (in the modern sense of know-how) and thought have parted company for good, then we would indeed become helpless’ [53].

## A life of a scientist is a life within a community

Putting the crystal ball to one side creates space for some personal self-reflection. As for a life of a scientist it is a simple truth (and it means happiness) that ‘no one walks alone’. Thus, I have to thank my wife Martina and my two sons, Alexander and Manuel, for their life-long patience, love, and support. In addition, I would like to mention a number of scientists who had markedly supported my development and added to my insights and understanding, thus, I would consider them as “brothers and sisters in spirit”; many thanks to Hans Joachim Seitz (Hamburg), Rolf-Dieter Hesch, Friedrich W Schmidt and Oliver Selberg (all from Hannover), Albert Burger (Geneva), Kevin Acheson and Yves Schutz (Lausanne), Steven B Heymsfield (Baton Rouge) and John Blundell (Leeds). During the last 20 years of my research activities Anja Bosy-Westphal (Kiel) had become my ‘researcher’s alter ego’, this was and still is a very close, intense, and sometimes bulky but always fruitful together where at the end the properties of ideas and concepts were and are left difficult to judge.

Being part of communities also concludes taking on duties. Accordingly, I have been a member of an endless number of committees and advisory boards. I also have a life-long experience as a reviewer for journals, funding agencies, and decision-makers including time-consuming activities in policy advice. In addition, I had spent some years as a president of the German Obesity Society and as the speaker of a big research network on obesity. Finally, I have 10 years of experience as an editor in chief of an international journal on clinical nutrition. Taken together, I consider all these duties as mixed experiences. Since I have never been interested to strive for power (and its common by-product getting money) I was not so well suited for some of those positions. Anyhow, to fulfill the jobs was more about balancing between the interests and the significances of other colleagues rather than about shaping a new and alternative world. Since scientists are no better people, there are similar problems in science as in other areas of our society. Thus, at the end, all these duties (although they are self-evident to do) also mean considerable losses of time and illusions.

## Should I recommend becoming a scientist to younger people?

Faced with the present big data research activities this question is difficult for me to answer. Since a scientist should find his or her own identity there is need of a definitive research question to be addressed within a cultural environment which promotes personal development rather than primarily increasing corporate profits. There should be no incentives to ‘produce’ science, this is also against a ‘publish or perish’ culture. A good answer to the question formulated in the heading could be, yes, if you really want to find out something. Or in other words, a certain mission consciousness is a precondition of becoming and being a scientist. Finally, a scientist should be free of some illusions and the pursuit of greatness while being aware of one’s limits. Taking that view we all should be aware, that the life of many valued colleagues tells us a more or less sad truth: While you can be a successful researcher publishing in the best journals and attaining the highest ordinations, most scientists will be forgotten within short-term. Anyhow, we should not worry about this. As Albert Camus has said, personal life is about understanding and reminder (from *Albert Camus, The Plague*). That’s enough value.
